# Translating Fibrosis to Malignancy: Biomarkers and Therapeutic Opportunities in Liver Fibrosis and Hepatocellular Carcinoma

**DOI:** 10.3390/medsci14010110

**Published:** 2026-02-25

**Authors:** Daniel Neureiter, Tobias Kiesslich, Matthias Ocker

**Affiliations:** 1 Institute of Pathology, Paracelsus Medical University, University Hospital Salzburg (SALK), 5020 Salzburg, Austria; d.neureiter@salk.at; 2 Cancer Cluster Salzburg, 5020 Salzburg, Austria; 3 Center of Physiology, Pathophysiology and Biophysics, Institute of Physiology and Pathophysiology Salzburg, Paracelsus Medical University, 5020 Salzburg, Austria; 4 Department of Internal Medicine I, Paracelsus Medical University, University Hospital Salzburg (SALK), 5020 Salzburg, Austria; 5 Medical Department, Division of Hematology, Oncology and Cancer Immunology, Campus Charité Mitte, Charité University Medicine Berlin, 10117 Berlin, Germany; 6 EO Translational Insights Consulting GmbH, 12247 Berlin, Germany; 7 Tacalyx GmbH, 12489 Berlin, Germany

**Keywords:** biomarker, cirrhosis, fibrosis, hepatocellular carcinoma, liver cancer, targeted therapies

## Abstract

Background/Objectives: Hepatocellular carcinoma (HCC) commonly arises from chronic liver diseases that show progressing fibrosis and cirrhosis. The molecular mechanisms driving the transition from advanced fibrosis to overt malignancy remain poorly defined, representing a key knowledge gap in current hepatology research. This review delineates shared pathways like TGFβ/SMAD, WNT/β-catenin, Hedgehog, NOTCH, Hippo/YAP-TAZ and MAPK, linking fibrosis to HCC and opening avenues for dual antifibrotic/antitumor therapies. Results and Conclusions: So far, validated biomarker tools for fibrosis, like FIB-4, Enhanced Liver Fibrosis (ELF) and combined direct/indirect markers of liver damage and tissue remodeling, are used for fibrosis staging, while HCC detection leverages serum parameters like α-fetoprotein (AFP) or, more recently, multi-omics approaches (miRNA, cfDNA, metabolomics). Understanding the interconnection of these pathways can lead to novel targeted therapies (e.g., TGFβ inhibitors) that may show dual antifibrotic and antitumor activity in future studies.

## 1. Introduction

Hepatocellular carcinoma (HCC) represents the most common type of primary liver cancer and is among the most common causes of cancer-related deaths worldwide [1]. The pathogenesis of HCC has been linked to chronic liver diseases, and metabolic and steatotic chronic liver diseases like metabolic dysfunction-associated steatotic liver disease (MASLD) and metabolic dysfunction-associated steatohepatitis (MASH) show rising incidence rates [2]. A pathophysiologic feature common to all chronic liver diseases (including viral hepatitis, alcohol abuse and MASLD) is the development of liver fibrosis, mediated by activation of hepatic stellate cells (HSCs), remodeling of extracellular matrix (ECM) and immune cell interactions, which, over time, result in cirrhosis and an increased risk of oncogenic transformation [3,4]. The global etiology of liver fibrosis and HCC is undergoing a major shift from direct viral oncogenesis to more metabolic and inflammatory drivers, with associated mitochondrial dysfunction and oxidative stress inducing liver fibrosis and cirrhosis with an associated increased risk of HCC [5,6,7]: MASLD and MASH, driven by metabolic factors like insulin resistance, adipose tissue dysfunction, and chronic low-grade inflammation, have become the most common chronic liver diseases worldwide. This rise is directly linked to the increasing prevalence of obesity and type 2 diabetes. While MASLD can progress through the typical fibrosis-cirrhosis sequence, a critical clinical challenge is that up to 30% of MASLD-related HCC cases occur in non-cirrhotic livers, which severely complicates standard surveillance and early detection strategies. Genetic variants, such as PNPLA3, TM6SF2, and HSD17B13, along with perturbations in the gut–liver axis, further modulate this individual risk [6,8]. The relevant molecular pathways of MASLD-related fibrogenic activation in the liver are associated with transforming growth factor-β signaling, Notch-induced osteopontin and sphingosine kinase 1-mediated responses [6]. In contrast, viral hepatitis (hepatitis B virus (HBV), hepatitis C virus (HCV)) remains a dominant global driver, particularly in Asia and Africa, though its relative contribution is declining due to universal vaccination programs and effective antiviral therapies. The oncogenic mechanisms of these viruses are distinct: HBV involves direct viral protein expression and host DNA integration, while both HBV and HCV trigger chronic inflammation and immune dysregulation [9,10]. Currently, infections still account for the majority of global HCC cases (65.9%), but this proportion is shrinking as metabolic risk factors (19.7%) rise. Alcohol-associated liver disease (ALD) remains a significant contributor (22.4%), characterized by direct hepatotoxicity, oxidative stress, and immune activation. Risk is influenced by dose, sex, and genetics, with the highest HCC risk concentrated in patients who have already developed cirrhosis [8,11]. Finally, cholestatic liver diseases (such as PSC and PBC), though less common, are increasingly recognized in Western countries. Driven by autoimmune and environmental factors, these diseases follow a distinct oncogenic trajectory, primarily increasing the risk for cholangiocarcinoma rather than HCC [12].

**Figure 1:**
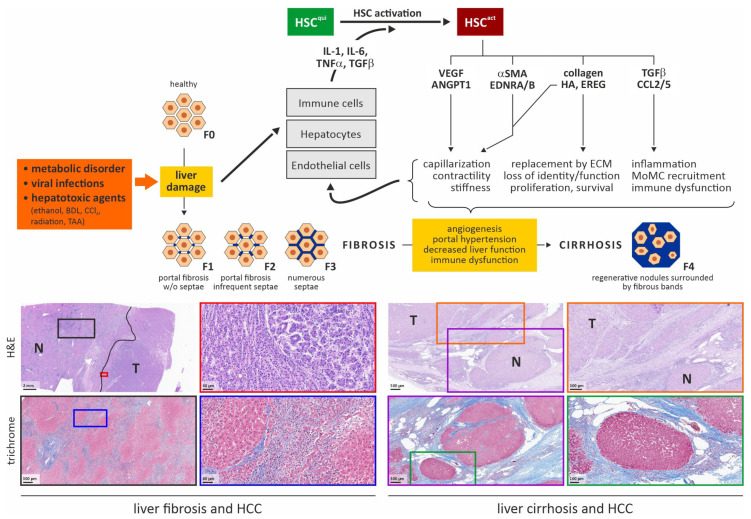
Pathomechanisms of liver fibrosis and progression to cirrhosis. The illustrated liver microarchitecture is categorized according to the METAVIR staging system, ranging from F0 (normal liver) to F4 (liver cirrhosis), depending on the extent of fibrotic deposition [38]. Conceptual framework adapted from [39,40,41]. The lower part shows two typical clinical cases of HCC arising within fibrotic (**left**) or cirrhotic (**right**) liver parenchyma visualized via H&E and Masson’s trichrome stain. For both panels, colored boxes indicate the areas shown in higher magnifications. Abbreviations: αSMA, α-smooth muscle actin; ANGPT1, angiopoietin 1; BDL, bile duct ligation; CCl4, carbon tetrachloride; CCL, CC-chemokine ligand; ECM, extracellular matrix; EDNRA/B, endothelin receptor A/B; EREG, proepiregulin; H&E, hematoxylin-eosin stain; HA, hyaluronic acid; HCC, hepatocellular carcinoma; HSCqui/act, quiescent/activated hepatic stellate cell; IL-1/6, interleukin1-6; MoMC, monocyte-derived macrophage; N, normal tissue; T, tumor; TAA, thioacetamide; TGFβ, transforming growth factor β; TNFα, tumor necrosis factor α; VEGF, vascular endothelial growth factor.

Congestive hepatopathy, parasitic infections such as *Clonorchis sinensis* or *Opisthorchis viverrini*, and exposure to environmental toxins like aflatoxin B1 (AFB1) each activate distinct profibrotic pathways that converge in promoting HSC activation and liver fibrosis. The underlying mechanisms involve CXCL9-mediated macrophage enrichment in congestive liver disease; ferroptosis-induced cytokine (IL-6 and TNF-α) release and TGFβ/Smad pathway activation in clonorchiasis [13]; and p53–PINK1/Parkin-dependent mitophagy, alongside extracellular vesicle-mediated communication between hepatocytes and HSCs following AFB1 exposure [14,15]. Persistent progression of these fibrotic processes is associated with an elevated risk of HCC, reflected by higher incidence in Fontan-associated liver disease; enhanced angiogenesis and acquisition of stemness features in C. sinensis-related HCC; and the synergistic DNA-damaging effects of AFB1 in the context of fibrosis [16,17,18]. As illustrated in Figure 1, the convergence of chronic insults—including metabolic dysregulation, viral infections, and hepatotoxic exposure—triggers sustained liver injury and consecutive activation of immune cells, hepatocytes and endothelial cells. A pivotal subsequent event in fibrogenic initiation and progression is the activation of hepatic stellate cells (HSCs). Consequently, persistent injury drives pathological remodeling of the sinusoidal endothelium, hepatocyte dysfunction, and the recruitment of diverse immune cell populations, culminating in the excessive deposition of the extracellular matrix (ECM). These structural alterations, defined by aberrant hepatocyte survival and proliferation, lead to clinically significant portal hypertension and hepatic insufficiency. Ultimately, this creates a protumorigenic microenvironment that facilitates the development of hepatocellular carcinoma.

Despite rapid progress in therapies for HCC [19,20]—including immune checkpoint inhibitors, targeted treatments, and emerging approaches like CAR T cell and RNA targeting therapies—the overall clinical success rate remains low [21,22,23,24]. This underlines the urgent need for systematic early detection and surveillance in high-risk fibrotic patients—where advanced fibrosis affects ~3.3% globally and drives >90% of HCC cases [25]—through integration of molecular (e.g., liquid biopsy and proteomic/genomic signatures) and clinical biomarkers, enabling personalized, pre-emptive interventions. HCC surveillance strategies boost early-stage detection, curative treatment, and survival, with 5-year mortality dropping from >70% (late detection) to <20% (early detection) [26,27].

Despite the identification of key oncogenic signaling pathways of HCC, the transition from fibrosis to malignancy remains incompletely understood [28]. Multiple reviews highlight that while genetic and epigenetic alterations, chronic inflammation, and microenvironmental changes are implicated in hepatocarcinogenesis, the precise mechanisms driving the progression from cirrhosis or advanced fibrosis to overt malignancy are still being elucidated [29]. It is widely believed that HCC develops in fibrotic livers as a result of chronic injury, which transforms HSCs into myofibroblasts. These activated myofibroblasts deposit a large number of ECM components, disrupting the structure of the liver, increasing tissue stiffness, and creating an environment that supports tumor formation [30]. Fibrosis not only alters tissue architecture but also actively encourages cancer development, as HSCs release pro-growth signals such as HGF, IL-6, PDGF, and Wnt ligands, which directly stimulate hepatocyte growth and survival. At the same time, the stiffened ECM boosts integrin signaling and traps growth factors, enhancing pathways like FAK/ERK/Akt/STAT3 and promoting the expansion of pre-cancerous hepatocyte clones [31].

Additionally, the fibrotic scar functions as a complex signaling hub. Changes in matrix components (like collagen I, III, and laminin) and their breakdown products trigger further inflammatory and cancer-promoting signals in both hepatocytes and liver progenitor cells. HSCs also alter the microenvironment by emitting VEGF and angiopoietins to support new blood vessel formation, producing chemokines that attract immunosuppressive cells (such as Tregs, MDSCs, and M2 macrophages), and expressing PD-L1, which weakens immune surveillance by NK/NKT cells—allowing tumors to evade the immune system during ongoing cycles of damage and repair [32].

Metabolic changes in HSCs, especially shifts toward glycolysis and glutaminolysis, help maintain their fibrogenic and tumor-promoting activity. Persistent inflammation, oxidative stress, and pressure from cell replication collectively fuel genome instability, epigenetic changes and, ultimately, the development of liver cancer [33].

Early detection of HCC continues to be a persistent clinical challenge, as most cases are diagnosed at advanced stages due to the insidious onset and limitations of current surveillance modalities and biomarkers [34]. The American Association for the Study of Liver Diseases (AASLD) does not recommend routine HCC surveillance for non-cirrhotic patients with HCV or MASLD, as the low annual incidence makes screening cost-ineffective and fails to identify a significant proportion of individuals at risk [35].

Recent studies have identified molecular events and signaling pathways, including dysregulation of transforming growth factor β (TGFβ), WNT/β-catenin, and ERK/MAPK axes, as well as epigenetic alterations like CDKN2A and RASSF1A methylation, that are commonly associated with HCC formation in fibrotic livers. Identification and validation of (non-invasive) biomarkers for liver fibrosis and HCC, such as serum scores (e.g., FIB-4), gene signatures, protein panels, cfDNA or miRNA signatures, are needed to transform risk stratification and surveillance strategies [36,37].

This article elucidates pathogenic pathways that bridge fibrosis and HCC formation, summarizes the current landscape of biomarkers, and highlights therapeutic opportunities poised to impact the outcomes of patients with liver fibrosis and HCC.

## 2. Common Pathways in Liver Fibrosis and Tumorigenesis

Chronic liver injury (largely independent of the underlying cause) leads to the development of a premalignant niche, characterized by persistent inflammation, oxidative stress, extracellular matrix remodeling and accumulation, and impaired tissue regeneration [3]. This niche favors the clonal selection and malignant transformation of hepatocytes, and potentially also of pluripotent stem cells. Interestingly, a limited set of signaling pathways has been reported to be involved in both fibrogenesis and tumorigenesis, indicating that HCC is the endpoint of a pathway-mediated process and not the result of spontaneous mutations [42]. In the following section, we will highlight some of the key pathways that are shared between fibrosis and liver cancer formation. It has to be noted that these pathways are persistently and simultaneously activated in different cell types involved in hepatic tumorigenesis, i.e., hepatocytes, hepatic stellate cells (HSCs), immune cells including macrophages (Kupffer cells) as well as progenitor cells. Along with processes related to chronic wound healing (tissue remodeling), genomic instability and an immunosuppressive tumor microenvironment, these pathways contribute to forming the premalignant niche in fibrotic liver tissues, making them interesting candidates for the development of biomarkers and targeted therapies [31,43].

Transforming growth factor β (TGFβ) is the key profibrotic cytokine [44,45]. It induces the SMAD-dependent transcription of ECM genes and promotes the transdifferentiation of quiescent HSCs into collagen-producing myofibroblasts [44,46,47,48]. Sustained TGFβ signaling under chronic disease conditions can also activate non-SMAD signal transduction pathways, including the PI3K/AKT [49,50] and ERK/MAPK [51,52] pathways. All of these pathways can induce and support HSC survival, epithelial-to-mesenchymal transition (EMT) and resistance to cell death pathways like apoptosis or ferroptosis, representing typical hallmarks of cancer [53]. One of the main challenges related to TGFβ-signaling remains in understanding why, when and how antitumor effects (e.g., cell cycle arrest and profibrogenic signaling) switch to protumorigenic effects, leading to invasion, angiogenesis and immune evasion [54,55,56], despite all of these processes being related to tissue remodeling.

While the TGFβ receptor is signaling through the SMAD pathway, other cytokine or growth factor receptors employ the PI3K/AKT or MAPK/ERK pathways, which also crosstalk with each other. While these pathways foster survival and proliferation of HSCs and activated myofibroblasts in fibrosis, they sustain proliferation and survival, metabolic reprogramming, and resistance to therapy [40,57,58]. Prominent examples of aberrant growth factor receptor signaling in both fibrosis and HCC are the FGF19/FGFR4 and HGF/c-MET pathways [59,60,61]. Upregulation of FGF19 and FGFR4 is strongly associated with progression from fatty liver disease through steatohepatitis and cirrhosis to HCC, with increased expression correlating with histopathologic severity and cancer stem cell markers, such as EpCAM [60]. In parallel, aberrant activation of the HGF/c-MET axis is found in both fibrotic and neoplastic liver tissue, with immunohistochemical studies demonstrating overexpression of HGF and c-MET proteins in cirrhosis and HCC, highlighting these pathways in both fibrogenesis and malignant transformation [62]. Targeting FGFR4 and c-MET signaling is under active investigation as a therapeutic strategy in HCC in relation to their roles in tumor proliferation, invasion, and progression [59].

Toll-like receptor 4, on the other hand, is activated by microbial products and endogenous danger signals and induces expression of pro-inflammatory cytokines via MyD88/IRAK1/IRKA4 (and MAPK) signaling or induces a type 1 interferon response via TRAM/TRIF signaling that could contribute to chronic inflammatory conditions in fibrosis and promote a protumorigenic microenvironment leading to HCC formation [40,63,64].

Several stem cell-associated signaling pathways have been implicated in both liver fibrosis and the development of HCC.

Under healthy conditions, WNT/β-catenin signaling is only weakly active in the adult liver. During chronic liver injury, however, it becomes reactivated and promotes the expansion of liver progenitor cells, activation of HSCs, and remodeling of the ECM [65,66]. Stabilized β-catenin accumulates in the nucleus and induces the transcription of genes that regulate cell proliferation, metabolism, and stemness. Notable targets include cyclin D1 and c-MYC, which are overexpressed during fibrosis and in HCC and help drive uncontrolled cell growth [58,59,60,61]. Additional β-catenin targets, such as LGR5, glutamine synthetase (GLUL), and AXIN2, are linked to progenitor cell activation and altered liver zonation, particularly when β-catenin is mutated [67,68,69,70,71].

Chronic liver damage also triggers the release of Hedgehog (Hh) ligands, including Sonic Hh, from injured hepatocytes. These ligands activate GLI-dependent transcription in HSCs and cholangiocytes, promoting ECM deposition, epithelial-to-mesenchymal transition (EMT), and angiogenesis [72,73].

Similarly, NOTCH signaling, which normally regulates biliary progenitor cell fate, becomes aberrantly activated in chronic liver disease. Engagement of the Jagged1-NOTCH pathway stimulates HSC activation and contributes to fibrosis. In HCC, NOTCH signaling is frequently upregulated and supports a progenitor-like, therapy-resistant, and EMT-associated phenotype [74,75,76].

Changes in the fibrotic ECM also influence mechanotransduction through the Hippo/YAP/TAZ pathway. Increased tissue stiffness and inflammation suppress Hippo kinase activity, leading to enhanced YAP/TAZ transcriptional activity in hepatocytes and myofibroblasts. YAP/TAZ target genes promote cell-cycle progression, ECM production, and cell survival [77,78]. In HCC, YAP/TAZ activation correlates with poor prognosis and a multi-drug-resistant phenotype [79]. The Hippo/YAP-TAZ pathway is a critical target within the liver fibrosis–carcinogenesis axis. However, this pathway exhibits context- and cell-specific effects, where its inhibition to reduce fibrosis can lead to divergent, uncontrolled adverse events and potential oncogenic risks [80]. Furthermore, there are currently no clinically validated Hippo/YAP-TAZ inhibitors available [81].

Several other factors also contribute to this complex and multi-layered signaling network that connects chronic liver disease and injury with inflammation and aberrant regeneration with fibrogenesis and ultimately hepatocarcinogenesis [82,83,84]. This provides the basis for a plethora of potential biomarkers and therapeutic targets for early treatment of liver cancer, already at the fibrotic stage.

The relevance of the aforementioned pathway is primarily based on preclinical evidence using in vitro and in vivo human cancer cells or genetically modified animal models [31]. However, as discussed in more detail in Section 5 (“Therapeutic opportunities to target liver fibrosis and HCC—focus on shared pathways”), initial and ongoing clinical trials—particularly those targeting TGFβ—align with preclinical findings derived from in vitro and in vivo models [85]. Interestingly, the TGFβ and Hippo/YAP-TAZ pathways are specifically associated with HCC risk stratification in the context of liver fibrosis. This makes them highly attractive targets from both a fibrogenic and carcinogenic perspective [47,77,80,86].

## 3. Fibrosis Biomarkers

The range of biomarkers used in liver fibrosis and HCC has moved beyond single, mostly nonspecific serum markers to multimodal panels that incorporate circulating proteins, ECM components, nucleic acids, metabolites, and imaging metrics. This transition responds to the growing demand for non-invasive methods to stage fibrosis, anticipate progression to cirrhosis, and identify and monitor those at risk for HCC development who may benefit from preventive therapies and earlier interventions. Notably, biomarkers with clinical relevance have been validated across patients suffering from viral hepatitis, alcohol-related liver disease, and MASLD, backing their translation into clinical practice with strong evidence [87,88,89].

The non-invasive assessment of liver fibrosis is based on composite scores that use routine laboratory tests (indirect markers) and ECM-related (direct) markers [90,91,92]. Indirect markers (e.g., aspartate aminotransferase, AST; alanine aminotransferase, ALT; platelet count; albumin levels, body-mass index, BMI) reflect hepatocellular dysfunction and metabolism, whereas direct markers, like hyaluronic acid, procollagen III N-terminal peptide (PIIINP) or tissue inhibitor of metalloproteinases-1 (TIMP-1), indicate active matrix deposition and tissue remodeling. Clinically commonly used scores are, e.g., APRI (AST-to-platelet ratio index), FIB-4 (age, AST, ALT, and platelets), NAFLD fibrosis score (NFS; age, BMI, diabetes/impaired fasting glucose status, AST/ALT ratio, platelets, and albumin), FibroTest/FibroSure (age, sex, a_2_-macroglobulin, haptoglobin, apolipoprotein A-I, g-GT, total bilirubin, and ALT), or the Enhanced Liver Fibrosis (ELF) test, which uses more direct ECM-related markers (HA, PIIINP, and TIMP-1). These scores have been validated against tissue biopsies and are usually specific for advanced fibrosis stages and for certain etiologies (e.g., hepatitis C virus-related or metabolism-related) [87,93,94,95]. On the one hand, indirect markers integrated into clinical scores (e.g., FIB-4 and APRI) are highly accessible and cost-effective. They offer a strong negative predictive value for ruling out advanced fibrosis, yet they are limited by indeterminate zones and reduced specificity in older patients or those with multiple comorbidities. On the other hand, direct markers (e.g., ELF Test and FibroTest) provide greater mechanistic specificity by reflecting actual extracellular matrix turnover, but their use is often constrained by higher costs, limited availability, and potential interference from extrahepatic factors. Consequently, the AASLD recommends a stepwise approach: initiating screening with indirect markers and reserving direct markers or imaging-based assessments for cases with indeterminate results or high clinical suspicion [96].

The current medical literature identifies FIB-4 as the most extensively validated and accurate non-invasive biomarker for predicting HCC risk across various liver disease etiologies. FIB-4 consistently outperforms other indirect markers, such as APRI and GPR, in forecasting HCC development. This is evidenced by superior area under the receiver operating characteristic curve (AUROC) values and higher hazard ratios for HCC incidence in patients with hepatitis B and/or C, as well as those with MASLD and alcohol-related liver disease [97,98,99,100].

Mechanism-based panels have recently also entered clinical validation. A panel comprising markers linked to collagen turnover (IGFBP7, SSc5D, and Sema4D) was derived from serum profiling in MASLD and outperformed FIB-4 in discriminating early (F0–F2) from late (F3/F4) fibrosis stages [95]. Other tests, e.g., LiverFASt and FibroMeter, combine direct and indirect markers and have shown high diagnostic accuracy superiority to FIB-4 and ELF in advanced fibrosis (F3/F4) and cirrhosis [101,102,103,104]. In summary, these scores enable fibrosis screening in broader patient collectives and long-term follow-up for cirrhosis and HCC monitoring without the need for invasive liver biopsies.

Emerging soluble biomarkers like the soluble triggering receptor, expressed on myeloid cells 2 (sTREM2) and glycoprotein non-metastatic melanoma protein B (GPNMB), show strong translational potential for non-invasive fibrosis assessment. sTREM2, shed from TREM2 and lipid-associated macrophages (LAMs), correlates with fibrosis stage even in early, asymptomatic chronic liver disease (AUROC 0.708) for predicting post-hepatectomy liver failure, outperforming the FIB-4/model for end-stage liver disease (MELD). It localizes to fibrotic areas, aiding early detection and prognosis in MASH/HCC progression [105]. GPNMB, which also marks LAMs alongside TREM2/CD9/CD14, reflects macrophage-driven fibrogenesis and resolves during fibrosis regression; its plasma levels predict inflammation in lysosomal storage diseases with liver involvement [106]. These macrophage-derived markers complement PRO-C3/PRO-C6 in dynamic monitoring, with clinical trials validating their prognostic utility in high-risk cohorts pre-HCC [107].

Functional imaging provides a complementary and further quantitative measure of fibrosis, as well as portal hypertension. Transient elastography (e.g., FibroScan) and magnetic resonance elastography estimate liver stiffness as a correlate to the histologic fibrosis stage. Cut-offs for clinically significant fibrosis (F2) and cirrhosis (F4) are now embedded in clinical guidelines, and longitudinal measurements have been shown to track the regression of fibrosis under antiviral therapies [108,109,110].

Innovative and translational animal models that recapitulate MASH–fibrosis–HCC development and human biology are the key to fully understanding the underlying pathophysiologic processes and developing novel biomarkers and treatment options. Besides classical diet-induced models like the choline-deficient diet or the Western diet (WD) (high fat, high cholesterol, and high fructose) [111], genetic models have recently gained attention. The MUP-uPA (Major Urinary Protein–urokinase plasminogen activator transgenic) mouse, when fed a high-fat diet, develops ER stress-induced hepatocyte damage, progressing to NASH-like pathology with ballooning, pericellular fibrosis, and spontaneous HCC (up to 85% incidence by 40 weeks), mirroring human steatohepatitic HCC [112]. The Foz/Foz (KLB^foz/foz^) mouse on WD exhibits hyperphagia-driven obesity, evolving through steatosis, MASH with neutrophil/monocyte inflammation, advanced fibrosis/cirrhosis, and HCC; it uniquely shows regression upon diet normalization, with preserved leptin signaling for fibrogenic fidelity [113].

These models enable single-cell profiling of HSCs and LAMs and evaluation of fibrosis dynamics and reversibility, outperforming chemical diets alone in clinical translatability and thus resemble human pathophysiology more closely.

## 4. HCC Biomarkers

α-fetoprotein (AFP) remains the most widely used biomarker for HCC, although its sensitivity is limited in early and small tumors. A combination of AFP with clinical parameters like age, sex and underlying etiology improves its performance, and scores like GALAD (gender, age, AFP, AFP-L3, and des-g-carboxy prothrombin (DCP)) were found to be superior to ultrasound alone in detecting HCC in populations at risk. AFP-L3 and DCP were associated with vascular invasion, poor differentiation, and recurrence after ablation or surgical resection and have thus been demonstrated to be independent diagnostic and prognostic biomarkers [36,114].

Several other markers have been explored in addition to these classical biomarkers. While some biomarkers (e.g., glypican-3 (GPC3), heat shock protein 70 (HSP70) and glutamine synthetase (GS/GLUL)) are recommended tissue biomarkers, others can be detected from serum specimens. Here, osteopontin, midkine, dickkopf-1, several cytokines and chemokines like IL-6 or CXCL10, as well as miRNA signatures, have shown encouraging results in early patient studies. Yet, most of these markers still lack validation across different etiologies and disease stages. Recently, multi-omics approaches, including metabolomics (e.g., FibraChek Dx, which measures taurocholic acid and L-tyrosine in serum), have also been suggested to be able to discriminate early HCC from cirrhosis [115,116,117,118]. The clinical utility of such scores has been shown by demonstrating the improved prognostic accuracy of a nine-parameter serum signature that includes growth factors, inflammatory mediators, and matrix-related proteins (IGF-1, IL-10, TGFβ_1_, adipsin, fetuin-A, IL-1β, macrophage stimulating protein α chain, serum amyloid A, and tumor necrosis factor α (TNF-α)) [116].

Liquid biopsies employing cell-free DNA (cfDNA) or circulating tumor DNA have been established as valuable prognostic and diagnostic tools in different cancer indications. While HCC is usually not linked to a distinct mutation, methylation patterns of certain genes (e.g., RASSF1A, GSTP1 and SEPT9) have been shown to be increased in cfDNA samples from patients with advanced fibrosis and HCC [118,119,120]. Furthermore, circulating miRNAs and exosomal RNAs (esp. miR-21, miR-122 and miR-224) have shown differential expression in fibrosis and HCC [121]. Yet, these panels need further standardization and validation in larger studies and from the real world [122].

Overall, clinically validated biomarkers for fibrosis, cirrhosis and HCC cover a vast range of parameters, ranging from assessing only fibrotic changes (FIB-4, ELF, and elastography) to tumor-specific markers (AFP, AFP-L3, DCP, GPC3, and methylated cfDNA). In between and evolving are mixed signatures covering fibrosis and oncogenic pathways. Further refinement and improvement will come from incorporating further clinical and patient-related parameters (etiology, genetics, and treatment response) into dynamic and longitudinal risk assessment models [123,124,125].

## 5. Therapeutic Opportunities to Target Liver Fibrosis and HCC—Focus on Shared Pathways

As outlined above, liver fibrosis and HCC share complex networks of signaling pathways related to chronic injury and inflammation, matrix remodeling and malignant transformation. Persistent profibrogenic stimuli lead to the activation of HSC and ECM deposition, which creates a premalignant niche supporting hepatocyte transdifferentiation, clonal expansion of progenitor cells, and an immunosuppressive microenvironment [30,31,126]. The key pathways and their main mediators have been outlined above. Compounds in clinical development targeting these pathways are listed in Table 1. Interestingly, although these compounds could potentially be used in a dual manner to simultaneously target fibrosis and HCC, study designs and endpoints do not allow for such a composite readout. Rather, studies investigating a compound in the fibrotic setting aim at parameters like liver function, liver stiffness or improvement in fibrosis grading, whereas HCC studies usually use survival or disease progression parameters as endpoints, and thus, these dual effects may not be captured for all compounds or clinical studies. A clinical proof of concept was obtained by antiviral therapies for HBV and HCV that promote fibrosis regression and reduce HCC risk by suppressing viral replication (and genomic integration for HBV) and inflammation. Nucleos(t)ide analogs like entecavir and tenofovir improve fibrosis in up to 74% of chronic HBV-infected patients after one year of treatment, with HCC risk halved (aHR 0.39). Direct-acting antiviral drugs (polymerase inhibitors, protease inhibitors, and NS5A inhibitors) achieve sustained virologic response rates of more than 95% in HCV, improving fibrosis in ~60% of cases and cutting de novo HCC risk by ~70% in cirrhotic patients, demonstrating that sustained viral control reverses early fibrosis and intercepts carcinogenesis [127,128,129,130].

Sorafenib and regorafenib—both approved multikinase inhibitors for advanced HCC—also exhibit properties in experimental models. They inhibit HSC activation, reduce ECM deposition, and suppress angiogenesis. In CCl_4_-induced fibrosis models, sorafenib alleviates liver scarring by inducing HSC ferroptosis through the HIF-1α/SLC7A11 pathway, and it lowers portal hypertension by downregulating angiopoietin-1 and limiting vascular remodeling [131,132,133]. Regorafenib shows comparable antifibrotic actions, decreasing angiogenesis in CCl4-induced and bile duct ligation models, as well as in portal vein obstruction, supporting its dual role in both fibrosis attenuation and HCC treatment [134].

Clinical data provide early evidence of similar effects in humans. In the REFINE study (enrolling 1005 patients with unresectable HCC and Child-Pugh A/B liver function), regorafenib demonstrated good tolerability and efficacy (median overall survival 13.2 months), together with indications of stabilized liver function and fewer portal hypertension-related complications in cirrhotic patients [135]. For sorafenib, a retrospective cohort of 17 patients with advanced HCC showed a significant reduction in liver stiffness—shear-wave velocity decreased from 2.37 to 1.90 m/s after 3 to 6 months—across both cirrhotic and non-cirrhotic groups, while serum fibrosis markers remained stable [131]. Another clinical analysis associated sorafenib use with improved portal venous hemodynamics in HCC patients with underlying cirrhosis, consistent with its preclinical antifibrotic mechanisms [136]. Together, these findings suggest that sorafenib and regorafenib may help slow progression along the fibrosis–HCC axis, providing a strong rationale for dedicated trials evaluating their antifibrotic potential.

The crosstalk between fibrogenic and oncogenic pathways provides opportunities for novel combination approaches. TGFβ can induce Hh and WNT pathway components, while Hh activation supports TGFβ signaling and HSC activation, thus creating auto-amplifying profibrogenic loops. In carcinogenesis models, WNT and Hh have been shown to cooperate, and dual inhibition leads to better antitumor efficacy in mouse models [137,138,139]. Such findings indicate that rational combinations may be able to reprogram the fibrotic tumor microenvironment and premalignant niche to a benign phenotype again. Optimized combinations could also be identified by further refining spatiotemporal and single-cell omics approaches [140,141]. It was recently shown that distinct subtypes of HSC maintain hepatocyte zonation through the modulation of WNT signaling via RSPO3, whereas others develop a protumorigenic phenotype with high TGFβ and chemokine expression [142,143].

**Table 1:** Selected drugs in clinical development with potential dual effects on fibrosis and HCC.

Drug Name	Mechanism of Action	Highest Clinical Phase	Key Outcomes	Comments	Year	References
Fibrosis-focused studies						
Pirfenidone	Inhibitor of p38 MAPK, and of TGFβ and TNFα synthesis	2 (liver fibrosis)	Improved liver stiffness measurement, liver function tests, QoL and MELD score in compensated cirrhosis	Approved for idiopathic pulmonary fibrosis	2025	[144]
Hydronidone	Derivative of pirfenidone	3 (HBV-related fibrosis)	Histologic improvement in fibrosis in combination with entecavir	Phase 3 ongoing	2023/2025	[145,146]
Pamrevlumab	α-CTGF mAb (IgG1)	2 (liver fibrosis)	No liver-related data published	Discontinued	2015 *	NCT01217632
Bexotegrast	Dual inhibitor of α_v_β_6_ and α_v_β_1_ integrins (TGFβ pathway inhibitor)	2 (liver fibrosis)	Reduced fibrosis markers (ELF, PRO-C3, MRI) relative to placebo at 12 weeks	FastTrack designation for PSC	2025	[147] NCT04480840
PLN-1474	Inhibitor of α_v_β_1_ integrin	1 (liver fibrosis, MASH)	Reduced liver fibrosis, steatosis and inflammation in mouse models; positive safety and PK profile in human healthy volunteers	Currently no development due to strategic decision and change in ownership	2025	[148]
Lixudebart	Claudin-1 mAb	1 (liver fibrosis)	Safety, PK and target engagement achieved in Ph 1 healthy volunteer study, ready for Ph 2	Preclinical data in PDX models confirmed antifibrotic effects	2022	[149] NCT05939947
Foscenvivint	Inhibitor of CBP/b-catenin complex formation (WNT inhibitor)	2 (cirrhosis)	Improvement in FibroScan, ELF score and hepatic collagen content	Preclinical antitumor effect in HCC models	2022/2020	[150,151]
HCC-focused studies						
Sorafenib	Multikinase inhibitor	Approved (HCC)	Inhibition of HSC activation, collagen synthesis and EMT in preclinical models	No dedicated antifibrotic trials	2014/2008	[131,133,136,152,153,154]
Regorafenib	Multikinase inhibitor	Approved (HCC)				
Vactosertib	TGFβ type I receptor kinase inhibitor	1 (HCC)	Preclinical data hint at potential use in liver fibrosis	Development prioritizes oncology development	2016	[155]
Galunisertib	TGFβ type I receptor inhibitor (ALK5)	2 (HCC)	mOS 17.9 months in combination with sorafenib	No further development due to cardiac safety related to Smad inhibition	2019	[156]
Focus on other indications, but with relevant read-outs						
Vismodegib	Smo antagonist	Approved (basal cell carcinoma)	Strong antifibrotic effects in preclinical models	Limited efficacy in oncology	2011/2020	[157,158]
Sotatercept	Activin type II receptor antagonist; bone morphogenetic protein 11 ligand inhibitor	Approved for Pulmonary Arterial Hypertension	No specific data on liver fibrosis, but mechanism of action is linked to HSC activation		2024	[159]
Cudetaxestat	Autotaxin inhibitor	2 (IPF)	Preclinical data hint at potential use in liver fibrosis		2022 *	NCT05373914
Montelukast	Leukotriene D4 antagonist	Approved for asthma	Preclinical data hint at potential use in liver fibrosis		2025	[160]

* Year at which the clinical trial was started. Abbreviations: CTGF, connective tissue growth factor; ELF, Enhanced Liver Fibrosis; EMT, epithelial-to-mesenchymal transition; HBV, hepatitis B virus; HCC, hepatocellular carcinoma; HSC, hepatic stellate cell; CBP, cAMP-response element-binding protein-binding protein; IPF, idiopathic pulmonary fibrosis; mAb, monoclonal antibody; MAPK, mitogen activated protein kinase; MASH, metabolic dysfunction-associated steatohepatitis; MELD, model for end-stage liver disease; mOS, median overall survival; MRI, magnetic resonance imaging; PDX, patient-derived xenograft; Ph, phase; PK, pharmacokinetics; PRO-C3, pro-peptide of type III collagen; PSC, primary sclerosing cholangitis; QoL, quality of life; TGFβ, transforming growth factor β; TNFα, tumor necrosis factor α.

## 6. Conclusions

Beyond canonical oncogenic signaling pathways, matrix-, stemness-, inflammation- and mechanotransduction-related pathways provide a critical interface and crosstalk between liver fibrosis and HCC. The convergence of well-characterized pathways—including TGFβ, Hedgehog (Hh), Wnt/β-catenin, MAPK/ERK, and mechanotransduction (YAP/TAZ)—elucidates the complex interplay between fibrosis and tumor formation. These pathways offer novel, albeit highly complex, therapeutic targets for early-stage intervention aimed at halting the progression from chronic liver disease to cirrhosis and HCC. In this context, the development of molecular biomarkers is crucial to identifying the initial shifts toward regenerative fibrogenesis. Detecting these early signals could allow for interventions that prevent transition to maladaptive reparative fibrogenesis and subsequent oncogenesis. Therefore, further studies are needed to further strengthen the argument that therapies normalizing matrix composition or mechanotransduction (i.e., alleviating fibrosis) may also lead to anticancer benefits in patients [31].

## Data Availability

No new data were created or analyzed in this study.

## Abbreviations

The following abbreviations are used in this manuscript:

AASLD	American Association for the Study of Liver Diseases
AFB1	Aflatoxin B1
AFP	α-fetoprotein
ALT	alanine aminotransferase
ANGPT1	angiopoietin
αSMA	α-smooth muscle actin
AST	aspartate aminotransferase
AUROC	under the receiver operating characteristic curve
BDL	bile duct ligation
BMI	body mass index
CCL	CC-chemokine ligand
CCl4	carbon tetrachloride
CTGF	connective tissue growth factor
cfDNA	cell-free DNA
DCP	des-γ-carboxy prothrombin
ECM	extracellular matrix
EDNRA/B	endothelin receptor A/B
ELF	Enhanced Liver Fibrosis
EMT	epithelial to mesenchymal transition
EREG	proepiregulin
GLUL	glutamine synthetase
GPNMB	glycoprotein non-metastatic melanoma protein B
GPC3	glypican-3
HA	hyaluronic acid
HBV	hepatitis B virus
HCC	hepatocellular carcinoma
HCV	hepatitis C virus
Hh	Hedgehog
HSC	hepatic stellate cells
HSCqui/act	quiescent/activated hepatic stellate cell
IL-1/6	interleukin1-6
IPF	idiopathic pulmonary fibrosis
LAM	lipid-associated macrophage
MASH	metabolic dysfunction-associated steatohepatitis
MASLD	metabolic dysfunction-associated steatotic liver disease
MΦ	macrophage
MELD	model for end-stage liver disease
MoMC	monocyte-derived macrophage
mOS	median overall survival
MRI	magnetic resonance imaging
MUP-uPA	major urinary protein-urokinase plasminogen activator transgenic
N	normal tissue
PIIINP	procollagen III N-terminal peptide
PDX	patient-derived xenograft
Ph	phase
PK	pharmacokinetics
PRO-C3	pro-peptide of type III collagen
PSC	primary sclerosing cholangitis
QoL	quality of life
sTREM2	soluble triggering receptor expressed on myeloid cells 2
T	tumor
TAA	thioacetamide
TGFβ	transforming growth factor β
TNFα	tumor necrosis factor α
VEGF	vascular endothelial growth factor
WD	Western diet
